# Surgical Treatment of Obstetric Plexus Lesions by Direct Coaptation Compared to Sural Nerve Graft Interposition

**DOI:** 10.1055/s-0044-1801398

**Published:** 2025-01-15

**Authors:** Justus Mai, Christa Kunigunde Raak, Thomas Ostermann, Jörg Bahm, Wolfram Scharbrodt

**Affiliations:** 1 Integrative Neuromedicine, Community Hospital Herdecke, Witten/Herdecke University, Herdecke, Germany; 2 Center for Integrative Medicine, Faculty of Health, School of Medicine, Witten/Herdecke University, Witten, Germany; 3 Chair of Research Methodology and Statistics in Psychology, Witten/Herdecke University, Witten, Germany; 4 Division of Plexus Surgery, Clinic for Plastic Surgery, Hand and Burn Surgery, RWTH Aachen University, Hospital Aachen Germany, Aachen, Germany

**Keywords:** obstetric brachial plexus palsy, direct coaptation, sural nerve graft, microsurgery

## Abstract

**Background** To date, there are no uniform guidelines for the treatment of obstetric plexus lesions in German-speaking countries. An end-to-end direct suture after resection of trunk neuroma is recommended for surgical treatment if tension-free coaptation is possible, whereas the use of autologous nerve grafts bridging the gap between the adaptation margins is advised by consensus if tension-free coaptation is impossible.

**Objective** The aim of the study was to investigate which reconstruction strategy may provide a better recovery of motor function for patients after obstetric brachial plexus lesion.

**Methods** This study compared postoperative functional outcome after obstetric brachial plexus palsy from a patient collective including a total of 43 children. The surgical techniques of plexus reconstruction by end-to-end coaptation versus the use of sural nerve interposition graft have been analyzed. Therefore, the degrees of active motion of abduction and external rotation in the shoulder joint, and flexion in the elbow joint were assessed using the neutral zero method.

**Results** For abduction in the shoulder joint, significantly better motor function was found in the group with direct sutures ( *p* = 0.033). For external rotation in the shoulder joint and flexion in the elbow joint, there was no statistically significant difference between the groups ( *p* = 0.284 and *p* = 0.270, respectively).

**Conclusions** This study could not demonstrate absolute superiority of either reconstruction method. Slight evidence was found for a better functional outcome for plexus reconstruction by direct coaptation. Further arguments support a better suitability of plexus reconstruction by direct suture if its use is justifiable.

## Introduction

Obstetric brachial plexus palsy (OBPP) is a serious complication of the vaginal birth process. Due to a size discrepancy between the newborn and the birth canal, shoulder dystocia and obstetric arrest with stretch injuries of the brachial plexus as a consequence may occur during the birth process. 1 2 3 These complications significantly impact the lives of the affected children. 4 5

The incidence of OBPP in industrialized countries is between 0.19 and 2.5 per 1,000 live births. 6 Despite the immense progress in modern medicine in recent decades, this figure has hardly changed. 7 Although neurological symptoms (paralysis, sensory disturbances) regress spontaneously in 66 to 92% of cases, 8 irreversible damages to the brachial plexus up to avulsion injuries of the nerve roots from the cervical spinal cord do occur. 3 9 10 In the absence of recovery of nerve function using conservative therapeutic measures, recent studies indicate a surgical brachial plexus reconstruction between the 6th and 9th month of life. 1 3

Different procedures are available for surgical reconstruction of the brachial plexus. A longitudinal incision in the lateral triangle of the neck above the clavicle provides surgical access above the anterior scalenus gap. After intraoperative exploration, the neuroma is excised and a nerve gap is created. A microsurgical plexus reconstruction follows, which can be performed intraplexically by autologous nerve grafts and/or extraplexically in the form of nerve transfers. In this case, the nerve endings can be coaptated by microsutures (8/0 to 10/0) as well as by fibrin glue. In autologous nerve grafting, the sural nerve is usually used as the donor nerve, the harvesting of which results in a defect (scar at the harvest site and loss of sensation in the innervated area). In case of complete plexus lesions including root avulsion, the stumps of C8 and Th1 can be transposed to the upper roots and fixed by direct suture under moderate tension. The stump of C7 is also frequently coaptated by direct sutures if the root is well preserved. Based on the good experience with this procedure, a surgical technique was developed to reconstruct upper (or extended upper) plexus lesions with narrow nerve gaps (<1.5 cm) by direct sutures under reasonable tension. 11

To date, there are no uniform guidelines for the treatment of obstetric brachial plexus lesions in German-speaking countries, but recommendations are integrated into the AWMF guideline for the treatment of peripheral nerve injuries. In the most recently published S3 guideline (06/2013), an end-to-end direct suture is recommended for the surgical treatment of peripheral nerve injuries with possible tension-free coaptation. If tension-free coaptation is impossible, the use of interposition devices to bridge the gap between the adaptation margins is unanimously recommended. 12 However, these recommendations are based on studies, some of which are very old, and their validity must be critically questioned in the light of the progress in microsurgery. 13 14

Therefore, this study aimed to investigate how successful neurological rehabilitation after obstetric plexus paresis with surgical reconstruction by direct coaptation is compared to surgical treatment by sural nerve interposition.

## Methods

### Intervention/Reconstruction Technology

Injuries to the brachial plexus were reconstructed either through direct suturing of the nerve ends or through sural nerve graft interposition. If the distance between the proximal and distal lesion edges was short enough (up to 1.5 cm), epineurial end-to-end sutures were used microsurgically under moderate tension to reconstruct the nerve strands. In cases where the gaps were too long (over 1.5 cm), nerve bundles were taken from the sural nerve intraoperatively and then used as interposition grafts to bridge gap.

### Patients/Recruitment Strategy

The patient collective included a total of 43 children with obstetric plexus palsy who underwent surgical reconstruction of the brachial plexus at the Euregio Reconstructive Surgery Unit of the Franziskushospital Aachen. Therefore, follow-up examinations of the patients were performed and the data archives of the Plexus Surgery Section of the Clinic for Plastic Surgery, Hand and Burn Surgery of the University Hospital Aachen and the Franziskushospital Aachen were used.

### Inclusion Criteria

All patients were included in which one of the two interventions/reconstruction techniques mentioned below was used exclusively. In addition, in order to generate the most accurate results possible, only children who had been operated on by the same senior surgeon (J.B.) were included in the study.

### Exclusion Criteria

Patients were excluded if both reconstruction techniques were used intraoperatively. Other criteria that led to exclusion from the study were:

- Time between surgery and follow-up <6 months.
- Patients whose legal guardians did not agree to participate in the study.
- Factors that make inaccurate determination of range of motion likely, such as language.
- Barriers or limited cooperation during the examination.
- Other preexisting conditions or developmental deficits that were not due to the obstetric brachial plexus injury.
- Subjects in whom both reconstruction strategies were combined or additional
- reconstruction strategies were used.

### Allocation

The patient population of 43 patients was divided into two groups, which differed in the surgical technique used. Group 1 ( *n* = 18) consisted of patients with plexus reconstruction achieved using only direct coaptation. Patients whose plexus lesions were treated entirely by means of interposition from the sural nerve were included in group 2 ( *n* = 25).

Group 1 data were collected in follow-up examinations, while group 2 data were taken from the documented follow-up examinations from the department's data archive.

### Outcomes

In the follow-up examinations, ranges of motion of the upper extremity were obtained according to the neutral zero method (0–180 degree system). The neutral zero method is a common measurement method for recording the extent of joint mobility in a standardized way. It is based on the neutral position, in which the body position is upright, the feet are closed, and the thumbs are directed forward. 15 In the neutral position, all directions of movement are specified as 0°. The goniometer is used to measure by how many degrees the neutral position can be left actively and passively in one direction of movement. 16

Technically, a measuring device (model: Tutoy Professional 360 degree Multi-Lineal Goniometer Angle Spinal Ruler-1PCS) was used to determine the range of motion of active shoulder abduction, active shoulder external rotation, and active elbow flexion. To record the examination results, the investigator first notes the two antagonized directions of movement and the final degrees of range of motion behind them. If the neutral position is exceeded, a “0°” is recorded in the center. An example of a physiological knee movement in the sagittal axis would be: knee right flexion/extension (140°/0°/10°). 13 If the neutral position cannot be achieved, the minimum deviation from the neutral position is noted in the middle. The movement mentioned first is noted as the final degree, the movement that is not possible is noted as zero degree. An example of such a pathological range of motion would be: knee left flexion/extension (100°/10°/0°). 16 In this case, full extension in the left knee joint would not be possible.

### Sample Size Calculation and Statistical Analysis

Due to the rarity of the disease, no estimate for the effect size could be determined from the existing literature. However, based on the current sample of 43 patients, an allocation ratio of 25/18, a power of 80%, and a level of significance of α = 0.05, the between group effect size should at least be above *d* = 0.9 to obtain statistically significant results.

Data were presented as percentages for nominal data and as median, mean, and standard deviation for metrically scaled data.

## Results

### Participants

A total of 43 patients were included in the study. Of these, 42% ( *n* = 18) underwent direct suture as a surgical reconstruction procedure, while 58% ( *n* = 25) were treated by sural nerve interposition. The median age at the time of surgery was 6.66 months (range: 2.16–24.2 months). Of the 43 study participants, 23 were female (53.49%) and 20 were male (46.51%). Obstetric plexus palsy was unilateral in all participants, and only the range of motion of the affected side was included. In terms of baseline characteristics, both study groups were comparable; baseline characteristics are shown in Table 1 .

**Table 1:** Baseline demographic and clinical characteristics of study participants

	Group 1		Group 2	
Sex	F = 9	M = 9	F = 14	M = 11
**Age at the time of surgery**	9.4 (SD = 4.19)		6.3 (SD = 3.75)	
**Period between surgery and follow-up**	40.5 (SD = 19.3)		47.5 (SD = 22.83)	

Abbreviation: SD, standard deviation.

**Fig. 1:**
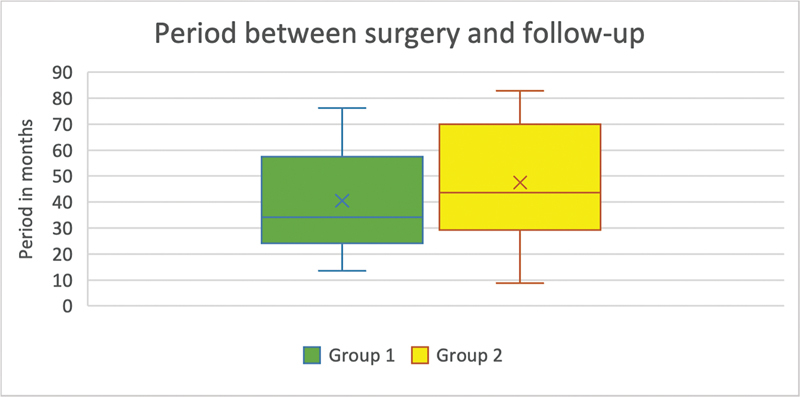
(Period between surgery and follow-up): On the *x* -axis are the two groups. On the *y* -axis, the time between surgery and follow-up is shown in months (group 1, *n* = 18; group 2, *n* = 25). In group 1, the mean was 40.5 months, the maximum was 76.1 months, and the minimum was 13.4 months. In group 2, the arithmetic mean was 47.5 months, the maximum was 82.8 months, and the minimum was 8.8 months.

For all subjects, a mean period of 44.5 months (standard deviation [SD] = 21.64 month) was found between the operation and the follow-up examination. In group 1, the average time between surgery and follow-up was 40.5 months (SD = 19.3 month). In 9 of 18 patients, the time from surgery to follow-up was between 24.8 and 57.5 months. The overall shortest period was 13.4 months, and the longest period was 76.1 months. In group 2, the mean time between surgery and follow-up was 47.5 month (SD = 22.83 months). In 50% of the patients, the time from surgery to the time of follow-up ranged from 29.2 to 70 months. Overall, in group 2, the shortest period was 8.8 months, while the longest period was 82.8 months ( Fig. 1 ).

### Comparison of Active Abduction and Elevation of the Shoulder

**Fig. 2:**
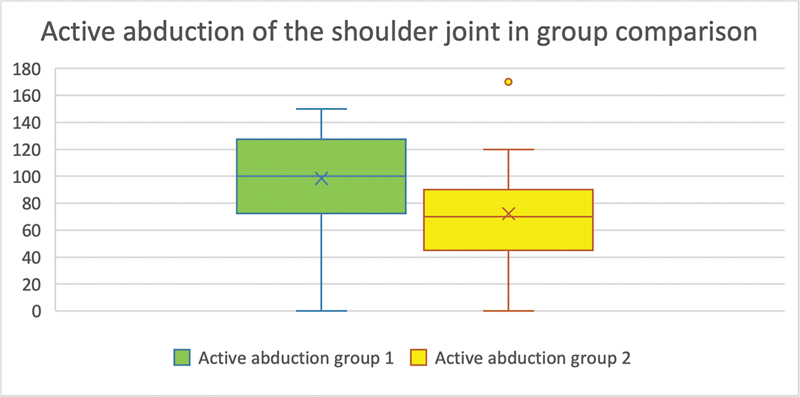
(Active abduction of the shoulder joint in group comparison): The *y* -axis shows the range of motion of the examined movement in degrees. On the *x* -axis are the groups compared with each other. The number of evaluable results in group 1 was 16 ( *n* = 16) and in group 2, 25 ( *n* = 25). In the case of active abduction in the shoulder joint, the degree of movement in group 1 was significantly greater than in group 2 ( *p* = 0.033).

In 16 patients of group 1 and in 25 patients of group 2, active abduction in the shoulder joint could be reliably determined. The mean value in group 1 was 98.438° (SD = 41.46°), and in group 2 the arithmetic mean was 72.2° (SD = 34.07°), which was significantly different between the groups ( *p* = 0.033; Fig. 2 ).

### Comparison of the Active External Rotation of the Shoulder

**Fig. 3:**
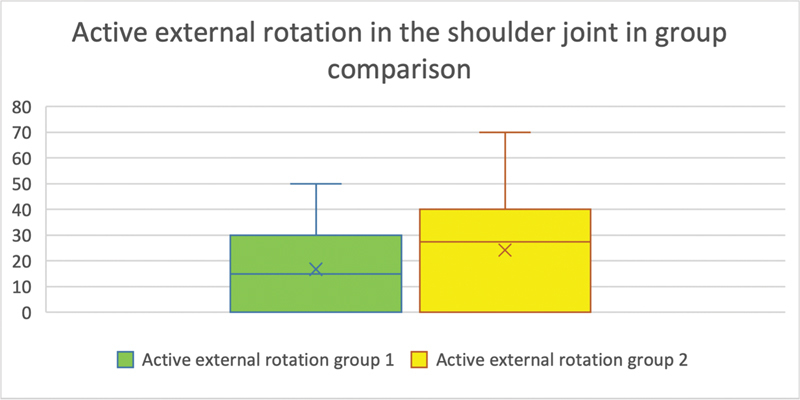
(Active external rotation of the shoulder joint in group comparison): On the *y* -axis is the range of motion of the examined movement in degrees. The *x* -axis shows the groups compared with each other. The number of evaluable results in group 1 was 17 ( *n* = 17) and in group 2, 18 ( *n* = 18). No significant difference in the degrees of movement between the groups could be detected ( *p* = 0.284).

The average range of motion in group 1 was 16.765° (SD =18.2). Eight of the subjects were unable to perform external rotation (range of motion = 0°). In group 2, the mean value was 24.167° (SD = 21.7°), with 6 out of 18 subjects showing a range of motion of 0°. There was no significant difference in the range of motion between the groups ( *p* = 0.284; Fig. 3 ).

### Comparison of Active Flexion in the Elbow Joint

**Fig. 4:**
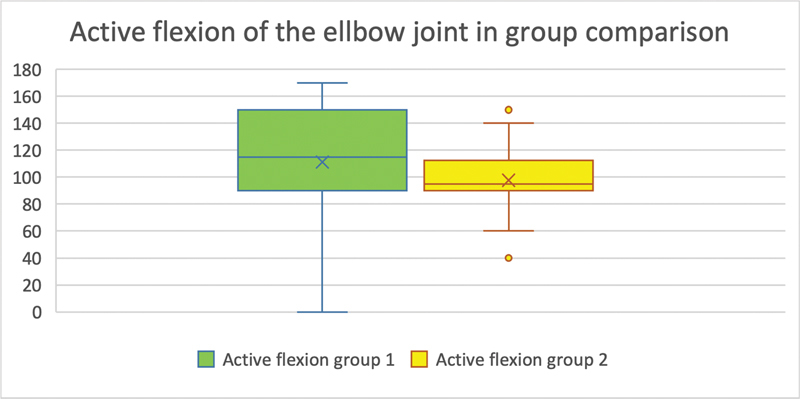
(Active flexion in the elbow joint in group comparison): On the *y* -axis is the range of motion of the examined movement in degrees. The *x* -axis shows the groups compared with each other. Only subjects with a reliable result were considered (group 1: *n* = 18; group 2: *n* = 22). No statistically significant difference between the degrees of movement in the active flexion of the elbow joint could be detected ( *p* = 0.270).

The range of motion in active flexion of the elbow joint was compared between the group in which direct sutures were used for surgical plexus reconstruction (group 1) and the group in which plexus was reconstructed by sural nerve interposition (group 2). The mean value of the ranges of motion in group 1 was 111.111° (48.98°), and in group 2 it was 97.727° (SD = 24.87°). Overall, the difference in ranges of motion in active flexion of the elbow between group 1 and group 2 was not statistically significant ( *p* = 0.270; Fig. 4 ).

## Discussion

To date, no uniform guideline exists for the choice of microsurgical procedure for plexus reconstruction after obstetric brachial plexus lesion. Millesi et al showed good results of nerve reconstructions by direct coaptation, as long as it was performed without tension in the early 1970s. 14 Based on this technique, tension-free reconstruction, mostly by graft interposition and/or nerve transfers, developed in the literature as a standard procedure. 10 17 However, in the recent literature, authors have presented direct coaptation under moderate tension as a reconstruction method that achieved encouraging postoperative motor results. 11 It is important to note that treatment of birth trauma to the brachial plexus is individualized and depends on many factors, including the severity of the injury and the age of the child at the time of injury.

### Active Abduction in the Shoulder

We were able to show that the range of motion achieved in active abduction in the shoulder joint could be approximately reproduced in the patients with direct sutures who were subsequently examined with a mean value of 98.4°, which is comparable with the results found in a previous publication, in which the authors found active abduction values of 75° after 6 months, 92° on average after 12 months, and 124° after 18 months. 11

A good motor recovery was also found for the patients treated with sural nerve interposition graft. The mean value for active abduction in the shoulder joint was 72.2°. Although we found statistically significant differences between the recorded ranges of motion for the respective groups, the difference cannot be interpreted as clinically meaningful. However, it can be seen as a strong argument for brachial plexus reconstruction by direct coaptation as a promising surgical procedure to be further explored.

### Active External Shoulder Rotation

For active external rotation of the shoulder after OBPP, the current literature is particularly concerned with the results of secondary interventions and corrective surgery.

In a meta-analysis, Louden et al found an improvement in active external rotation of the shoulder for both arthroscopic and open soft-tissue shoulder surgeries. 17 The same conclusion was reached by McKellar et al. In a systematic review, the authors described the improvement of the structural and functional characteristics of the affected shoulder by secondary corrective surgery. In addition, they also found a range of motion averaging 48° in active external rotation. 18 In contrast, the range of movements determined in this study is somewhat inferior. The high number of patients in whom active external rotation in the shoulder joint was not possible was striking (group 1: *n* = 8, 44.4%; group 2: *n* = 6, 24%).

The explanation for this fact is thought to be the failure to account for any secondary corrective surgery.

Overall, due to the same factors as for active abduction, the results obtained must be discussed critically with regard to their informative value for the question of the choice of the best surgical reconstruction strategy. Small numbers of cases, individually different injury morphologies, and the resulting very heterogeneous movement restrictions do not allow any absolute conclusions. In addition, due to the lack of statistical significance, none of the surgical methods seems to be favorable ( *p* = 0.284).

Considering the literature review in combination with the results of our study, the optimal outcome seems to depend less on the primary reconstruction method chosen. Rather, the choice of the most appropriate secondary corrective surgery as well as the best time to perform it plays a central role in the long-term outcome of active external rotation, as also described by other authors. 18

### Active Elbow Flexion

Active flexion in the elbow joint is highlighted as a particularly relevant prognostic factor and its restitution as one of the most important goals in microsurgical plexus reconstruction. 19 20 In a meta-analysis, the authors compared recovery of motor function after microsurgical plexus reconstruction between autologous nerve grafting and nerve transfers.

The results were comparable to the data collected in this study.

Thus, in Tora et al, 96.4% ( *n* = 54/56) of patients with nerve grafts and 95.2% ( *n* = 59/62) of patients with nerve transfers met the established criteria for adequate recovery of motor function (Medical Research Council score ≥ 3 or Active Movement Scale ≥ 5). 20 The results were confirmed in the present patient collective for patients with sural nerve interposition. However, good postoperative results according to the criteria of Tora et al could also be found for the patients with direct sutures.

Bahm et al measured for elbow flexion after reconstruction by direct coaptation under moderate tension movement radii between 60° and 90°. 11 The upper limit of this range was also reached by patients with sural nerve interposition (average 97.73°). For the patients who were treated with direct sutures, the mean range of motion was even 111.11°. Thus, in this comparison of the patient collectives, elbow flexion showed greater ranges of motion in the patients whose plexus lesions were treated with direct sutures. However, the difference did not show statistical significance ( *p* = 0.270). It can be concluded that plexus reconstruction by direct sutures for elbow flexion may be a promising surgical method. However, whether direct coaptation actually allows better motor recovery could not be clearly answered in this study despite discretely better range of motion, not least because of the lack of statistical significance.

### The Nerve Donor Defect

In autologous nerve transplantation, a nerve donor defect is unavoidable during the removal of the donor nerve. This consists not only of wound formation and subsequent scarring, but also the loss of sensitivity in the skin area supplied by the donor nerve. 6 11 The complications of sural nerve harvest are well described. They include, in descending frequency, chronic pain, paresthesia, cold perception disorder, and noninfectious and infectious wound healing disorder, in addition to loss of sensation. 21 22

Overall, the expected recovery of plexus function should be the main decision criterion. However, the harvesting of the donor nerve is associated with some not inconsiderable risks and complications. Therefore, with an otherwise equivalent expected outcome, it provides another argument against the use of autologous nerve grafts.

### Limitations

Due to the retrospective nature of the work and the rarity of the disease, the number of patients included in the study was small and unequal between groups, which clearly affected statistical significance. Moreover, the mentioned individuality and variance of obstetric plexus paresis further limited the comparability of the study participants among themselves. Furthermore, postoperative factors such as regular physiotherapeutic treatment or secondary corrective surgery were not included in the categorization of the participants. For better comparability, recovery of motor function was used in this study as a parameter for postoperative outcome. However, the multidimensionality of the limitations of patients with obstetric plexus paresis cannot be adequately reflected by the evaluation of motor recovery alone. 23 24 Finally, a longer follow-up period would allow a better assessment of postoperative range of motion.

## Conclusion

In summary, our study did not demonstrate an absolute advantage for postoperative outcome with any of the two surgical methods. We found evidence for a better suitability of plexus reconstruction by direct sutures if their use is well justifiable intraoperatively. Thus, the use of interposition sutures for large gaps between adaptation margins remains indisputable. Prospective studies examining intraoperative conditions such as tension and defect distance, as well as using more comprehensive methods to survey the postoperative situation, should be conducted. This could strengthen the position of end-to-end coaptation as a surgical method.
